# Design of stimuli-responsive minimalist heptad surfactants for stable emulsions

**DOI:** 10.1038/s43246-024-00670-6

**Published:** 2024-10-15

**Authors:** Yang Li, Yilun Weng, Yue Hui, Jiaqi Wang, Letao Xu, Yang Yang, Guangze Yang, Chun-Xia Zhao

**Affiliations:** 1 School of Chemical Engineering, Faculty of Science, Engineering and Technology, The University of Adelaide, Adelaide, SA 5005 Australia; 2 ARC Centre of Excellence for Enabling Eco-Efficient Beneficiation of Minerals, Adelaide, SA, Australia; 3 Australian Institute for Bioengineering and Nanotechnology, The University of Queensland, Brisbane, QLD 4072 Australia; 4 Wisdom Lake Academy of Pharmacy, Xi’an Jiaotong—Liverpool University, Suzhou, Jiangsu 215123 China

**Keywords:** Colloids, Soft materials

## Abstract

Peptide surfactants have been extensively investigated with various applications in detergents, foods, and pharmaceutics due to their biodegradability, biocompatibility, and customizable structures. Traditional peptide surfactants are often designed in a head-to-tail fashion mimicking chemical surfactants. Alternatively, a side-by-side design pattern based on heptad repeats offers an approach to designing peptide surfactants. However, minimalist peptide design using a single heptad for stabilizing interfaces remains largely unexplored. Here, we design four heptad surfactants (AM1.2, 6H, 6H7K, and HK) responsive to metal ions and compare their emulsification performance with a three-heptad peptide, AM1. Among them, the HK peptide generates emulsions exhibiting good stability over months. We further optimize factors such as buffering salts, ionic strength, and emulsion dilutions to uncover their impacts on emulsion properties. Our findings deepen the understanding of emulsion properties and provide practical insights for characterizing peptide-based emulsions, paving the way for their broader utilization in diverse applications.

## Abstract (web summary)

Peptide surfactants are useful in detergents, foods, and pharmaceutics but their design using a single heptad remains largely unexplored. Here, four heptad surfactants were designed that are responsive to metal ions and show good emulsification properties.

## Introduction

Surfactants have been widely applied in many fields such as household detergents, pharmaceutics, and biosensing^1–3^. For example, water-soluble vitamin E (α-tocopheryl poly(ethylene glycol) succinate) has been employed as a contrast agent for medical ultrasound imaging to stabilize microbubbles^4,5^, and anionic alkylbenzene sulfonate has been commonly used as a household detergent^6^. These surfactants adopt a head-to-tail structure consisting of a short hydrophilic head linked to a long hydrophobic chain^7,8^. However, many traditional chemical surfactants pose environmental and health challenges due to their persistence and toxicity^9^.

Peptide surfactants offer promising alternatives due to their biodegradability, biocompatibility, tunable structures, and functionalities^10,11^. The modular building blocks known as amino acids can be classified into polar and non-polar groups based on their hydrophobicity. In general, the structure of peptide surfactants can be categorized into two design patterns according to the allocation of hydrophobicity regions: (1) end-by-end (head-to-tail), also known as surfactant-like peptides, which comprises a continuous chain of hydrophobic amino acids typically ending with one or a few charged hydrophilic amino acid; and (2) side-by-side, consisting of alternating hydrophilic and hydrophobic amino residues in the peptide chain^8^. By engineering the peptide sequence and length, it is feasible to attain specific secondary structures such as α-helices or β-sheets, leading to unique properties including self-assembly^12^, stimuli-responsive activity^13^, and antibacterial effects^14^. Consequently, these peptide surfactants have been extensively explored. For example, Ac-A_6_K can effectively stabilize G protein-coupled receptor bovine rhodopsin^15^. DKDKC_12_K and DKDKC_12_D improve solubilization of photosystems I and II of cyanobacteria^16^. A heptad is a structural motif in the formation of α-helical structures^17^. It consists of seven amino acids, *a*, *b*, *c*, *d*, *e*, *f*, and *g*, with *a* and *d* positions being hydrophobic amino acids, and *b*, *c*, and *f* being hydrophilic amino acids^13^. One α-helical turn comprises 3.7 amino acids with a vertical unit of translation of 1.47 Å per residue^18^. Short heptads with helical structures capable of self-assembling into supramolecular nano assemblies have been explored by introducing the non-coded amino acid, α-aminoisobutyric acid (Aib)^19^. A single heptad SHR-FF (H_2_N-S-Aib-F-S-Aib-F-Aib-OH) was designed to form helical fibrillar assemblies and maintain residual helicity at 90 °C. Moreover, peptide surfactants such as SHR-FLLF (H_2_N-F-Aib-LA-Aib-LF-OH) and SHR-FLEFL (H_2_N-F-Aib-LE-Aib-LF-OH) were able to stabilize 20% silicone oil in water emulsions for over two months^20^.

We previously designed a three-heptad peptide, AM1 (Ac-MKQLADS-LHQLARQ-VSRLEHA-CONH_2_)^21,22^ that is capable of transforming into an α-helical structure from a random coil structure upon its absorption at interfaces^23^, with hydrophilic amino acid residues (K, D…) facing the hydrophilic phase, and hydrophobic residues (M, L, A…) oriented towards the hydrophobic phase. In addition, AM1 peptide forms stable foams and emulsions due to the formation of a film structure at the interface as a result of cross-linking between the metal ions (e.g. Zn^2+^) to histidine (His) in the peptide^22,24^. Furthermore, the stability of emulsions or foams can be modulated by pH or chelating agents to switch on or switch off the film state. This unique stimuli-responsive behavior expands the potential of AM1 peptides for various applications.

We demonstrated the excellent surface activity of the three-heptad peptide AM1. However, the question remains whether a minimal heptad, or single heptad, employing naturally occurring amino acids still exhibits surface activity, and how different environmental conditions or stimuli affect their capability in stabilizing the interface. In addition, many studies have shown that the addition of salts such as NaCl, CaCl_2_, and KCl destabilizes peptide-based emulsions^25–29^. In this study, four single-heptad peptides were designed and evaluated for their surface activity, and emulsification capability as well as the impact of environmental conditions such as buffer solutions and ionic strength. By employing a single heptad, it provides simpler synthesis, easier characterization, and lower cost of production that could expand the scope of applications of peptide surfactants.

## Results and discussion

### Peptide design and their emulsification capabilities

**Fig. 1:**
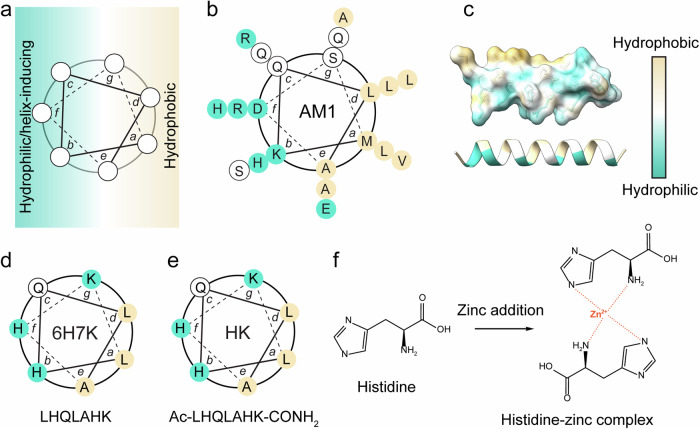
Peptide design. Schematic diagram of (**a**) design rationale of α-helical peptide. **b** Design and sequence of AM1 peptide, and **c** animated diagram of AM1 structure at the interface. **d**, **e** Design rationales for 6H7K and HK heptads. **f** Illustration of His-zinc coordination upon zinc chloride addition to the peptide emulsion system. Hydrophobicity was labeled in colors from green to white to yellow as hydrophilic to hydrophobic. ChimeraX was employed to generate the visualization of AM1 peptide structure in (**c**)^50^.

The peptide design was inspired by a side-by-side structure where hydrophobic amino acids are located at *a* and *d* positions, and hydrophilic amino acids at *b*, *c*, and *f* positions (Fig. 1a). Four heptad surfactants (summarized in Table 1) were designed based on AM1 consisting of three heptads (Fig. 1b). At an interface, AM1 is capable of forming a side-by-side structure conferring its surface activity (Fig. 1c). AM1.2 (LHQLARQ) is the middle heptad from the AM1 sequence consisting of only one His. The heptad 6H (LHQLAHQ) was designed by substituting arginine (Arg) with His to enhance His-zinc coordination. Under neutral conditions, the 6H peptide has a theoretical isoelectric point (pI) of 6.95. To investigate the charge effect, the amino acid glutamine (Gln) at the C-terminal of the 6H was replaced with lysine (Lys) to enhance the net charge of the peptides at neutral pH, resulting in the creation of heptad 6H7K (LHQLAHK) as illustrated in Fig. 1d. To further investigate the effects of terminal modification, both free ends of 6H7K were capped to form HK (Ac-LHQLAHK-CONH_2_) in Fig. 1e. The His-zinc coordination is demonstrated in Fig. 1f, in which two His residues are capable of interacting with one free Zn^2+^.

**Table 1:** Peptide sequences and theoretical pI for AM1, AM1.2, 6H, 6H7K, and HK

Peptide No.	Surfactant	Peptide sequence	Theoretical pI	Net charge at pH 7.5
1	AM1	Ac-MKQLADS-LHQLARQ-VSRLEHA-CONH_2_	10.99	+ 1.07
2	AM1.2	LHQLARQ	9.99	+ 0.51
3	6H	LHQLAHQ	6.95	− 0.45
4	6H7K	LHQLAHK	8.90	+ 0.55
5	HK	Ac-LHQLAHK-CONH_2_	N/A	+ 1.07

pI values were sourced from Prot pI^49^

**Fig. 2:**
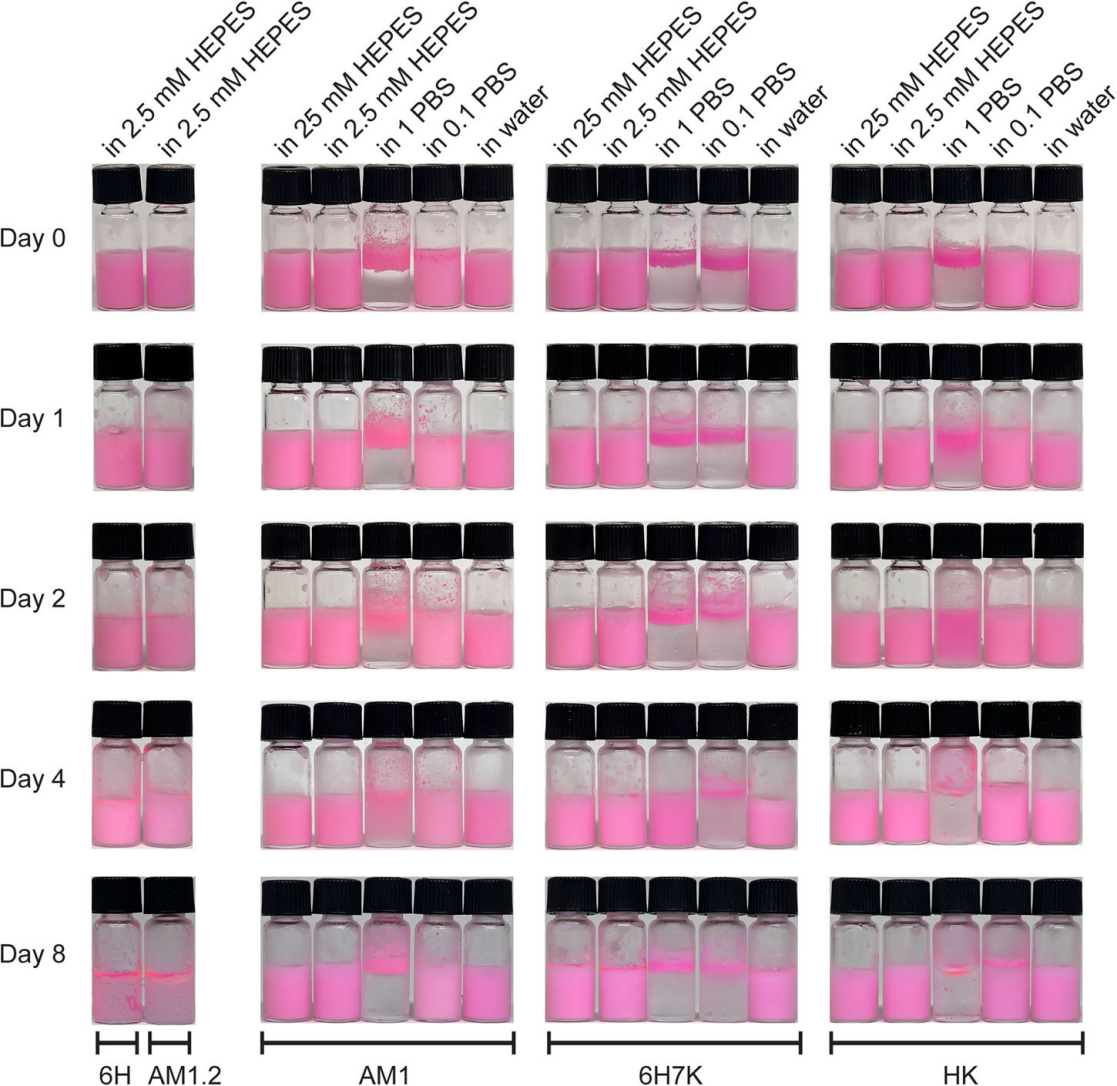
Images of emulsions over eight days. Images of emulsions stabilized by 6H and AM1.2 in 2.5 mM HEPES buffer; AM1, 6H7K, and HK in 25 mM HEPES, 2.5 mM HEPES, 1 × PBS, 0.1 × PBS, and water with a heptad concentration of 800 µM, AM1 concentration of 267 µM, and zinc chloride at twice the concentration of the peptides. DiI stain was added in the oil phase to help visualize the phase separation.

Preliminary studies on the four peptides AM1.2, 6H, 6H7K, and HK were conducted to generate 2% oil-in-water emulsions, using AM1 as a benchmark, and characterize their properties such as size, charge, and stability. A constant heptad concentration of 800 µM and AM1 concentration of 267 µM were adopted with two times concentrated zinc chloride at pH 7.5 in 2.5 mM HEPES buffer. DiI was used to label the Miglyol 812N (Mig 812 N) oil phase to help visualize the dispersion of emulsion, demonstrating insignificant effects on emulsion properties as illustrated in (Supplementary Fig. 1). All the peptides were able to make emulsions. AM1.2 and 6H emulsions showed clear phase separation on Day 8 (Fig. 2). In contrast, the emulsions formed by HK and AM1 remained stable without signs of separation throughout the first eight days while 6H7K emulsions had a slight phase separation on Day 8. The primary reason for the instability of AM1.2 emulsions is the presence of only one His residue, which is insufficient for forming His-zinc coordination bonds. On the contrary, 6H lacks charged amino acids resulting in a neutral theoretical pI of 6.95. Thus, the poor stability of the emulsions was mainly due to the lack of electrostatic repulsion at pH 7.5. Consequently, further investigation was not pursued for AM1.2 and 6H.

To explore the influence of buffer types and concentrations on emulsion stability, four different buffer solutions including 1× and 0.1× PBS, 25 mM and 2.5 mM HEPES buffer, and water were investigated. As expected, AM1, 6H7K, and HK failed to stabilize emulsions in PBS buffering system. Although, HK heptad has a lower interfacial tension in 1 × PBS, an immediate coalescence was observed, which was attributed to the elevated salt concentration in PBS screening the charge (Supplementary Fig. 2). At 1× PBS, emulsions aggregated and floated to the top immediately after being generated and giving a size of above 4000 nm due to the low net charge of below 5.3 mV. Therefore, it is important to incorporate other stabilization mechanisms in addition to electrostatic repulsion when designing surfactants. In contrast, emulsions of AM1 and HK remained stable in 25 mM and 2.5 mM HEPES buffer, and water throughout eight days, while 6H7K emulsions displayed slight phase separation in all buffer conditions. Subsequently, the following experiment was conducted in HEPES buffer and water for AM1, 6H7K and HK heptads.

The ionic strength of the surrounding environment also plays a crucial role in peptide emulsion stability. Several studies have shown that adding salts like NaCl, CaCl_2_, and KCl destabilized emulsions formed by peptides^25–28^. This destabilization arose from the screening effect of these ions, which reduced the electrostatic repulsion between peptides at the oil-water interface^30^. Additionally, buffering ions could further decrease peptide net charge by adsorbing onto their surface leading to a decline in electrostatic stability^31^. As a result, emulsion droplets become more susceptible to coalescence. For example, standard PBS buffer consisting of 137 mM NaCl, 2.7 mM KCl, 10 mM Na_2_HPO_4_, and 1.8 mM KH_2_PO_4_, exerts a strong screening effect resulting in the instant coalescence of emulsions (Fig. 2). Conversely, emulsions prepared in 25 mM, 2.5 mM HEPES, and water exhibited better stability due to the weaker screening effect of these buffering salts.

### Peptide structures in bulk solution and at interface

**Fig. 3:**
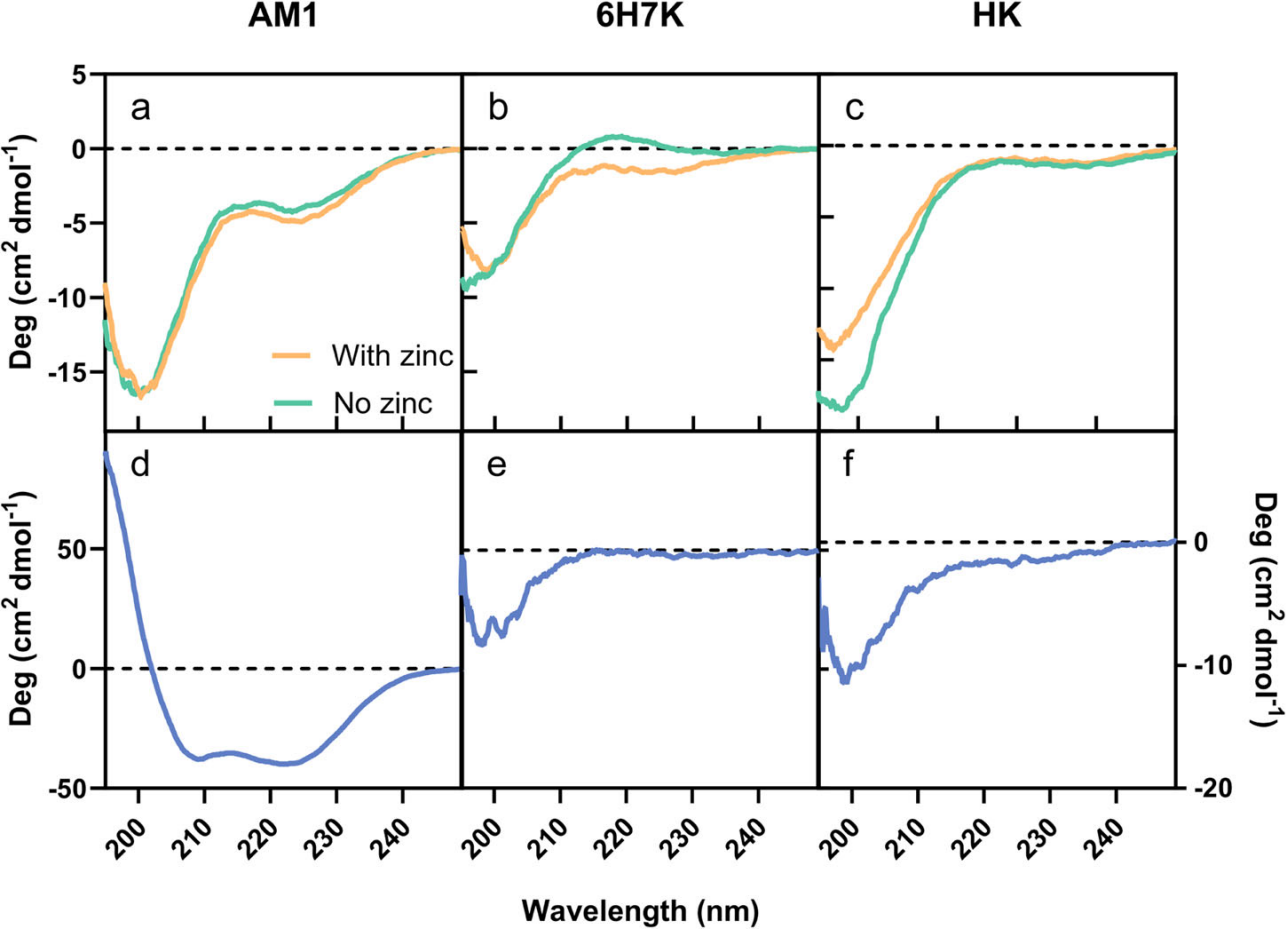
Circular dichroism results of peptides AM1, 6H7K, and HK. **a**–**c** In bulk with zinc (yellow) and without zinc ions (green), and **d**–**f** at the interface.

Circular dichroism has been widely applied in studying the secondary structure of peptides and proteins in bulk solutions^32–34^. The structures of AM1, 6H7K, and HK peptides in bulk solution were investigated in the presence and absence of zinc ions. All three peptides exhibit predominantly random coil structures (Fig. 3a–c).

**Fig. 4:**
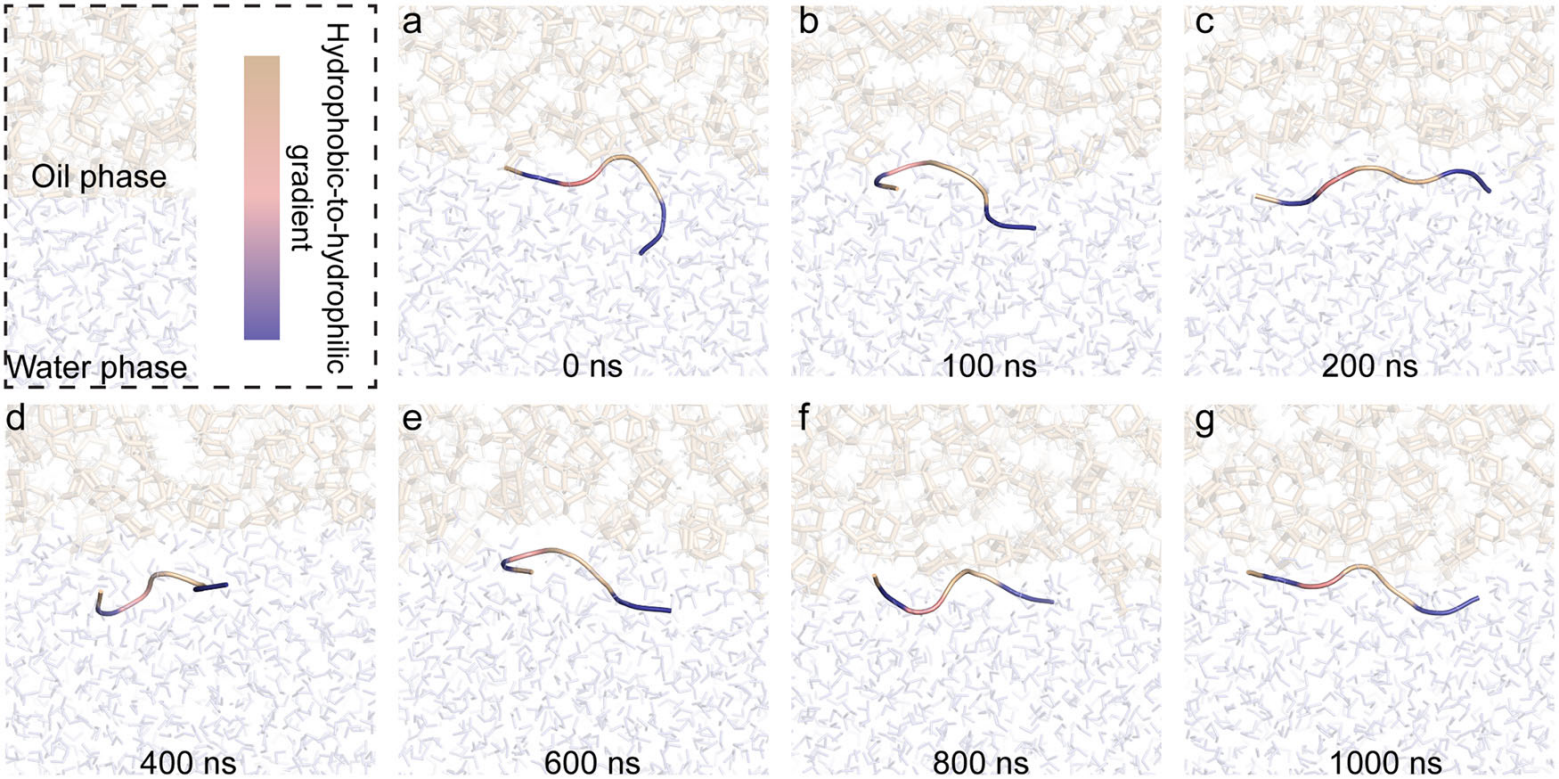
Molecular dynamics simulation of HK structures at Oil/Water interface for 1000 ns at pH 7.5. The structure was plotted out via Pymol at **a** 0 ns, **b** 100 ns, **c** 200 ns, **d** 400 ns, **e** 600 ns, **f** 800 ns, and **g** 1000 ns.

Determining peptide structures at oil/water interfaces is challenging due to differences in the refractive indices (RI) of two different liquid phases. Inspired by previous works^32,35,36^, glycerol was used to adjust the RI of the aqueous phase to match that of the oil phase. To minimize the required volume of glycerol for RI matching, Mig 812 N was replaced with silicone oil as it has a lower RI (1.403) than that of Mig 812 N (1.450). As a proof of concept, the structure of AM1 peptide at the oil-water interface was measured using CD (Circular Dichroism Spectroscopy), revealing a helical structure previously confirmed by neutron reflectometry and dynamics simulation^23^ (Fig. 3d). A similar approach was then applied to study the structure of heptads at the interface. The heptads primarily exhibit random coil structures at the interface (Fig. 3e, f). To further illustrate their structures, a molecular dynamics (MD) simulation was conducted at the oil/water interface for 1000 ns (Fig. 4). It can be observed that the HK peptide arranges in a side-by-side orientation at the interface, driven by the allocation of amino acids that positions hydrophilic residues toward the water phase and hydrophobic residues toward the oil phase.

### Surface activity

**Fig. 5:**
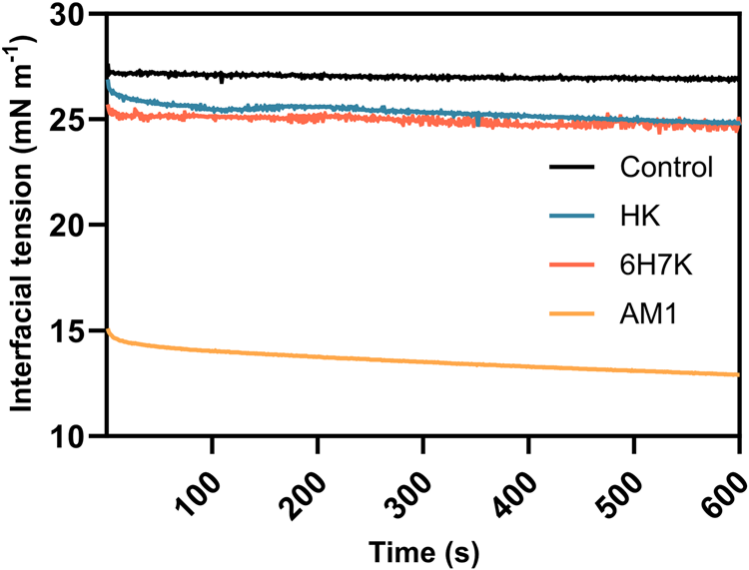
Comparison of dynamic interfacial tension of peptides. A Mig 812 N oil droplet was allowed to stabilize in AM1 (in orange), 6H7K (in red), and HK (in blue) solution with a peptide concentration of 100 µM and zinc chloride concentration of 200 µM. The control (in black) was measured at the oil/water interface of Mig 812 N oil and 2.5 mM, pH 7.5 HEPES buffer.

The interfacial tension of Mig 812 N oil in 100 µM AM1, 6H7K, and HK solutions in the presence of 200 µM zinc chloride was measured using a solution containing 2.5 mM HEPES and 200 µM zinc chloride at pH 7.5 as the control that has a surface tension of 26.97 mN m^−1^. Among all, AM1 exhibited the lowest interfacial tension of 12.90 mN m^−1^, followed by HK (24.83 mN m^−1^) and 6H7K (24.77 mN m^−1^) (Fig. 5) demonstrating the good surface activity of AM1.

### Metal ion responsive design based on His-zinc coordination

**Fig. 6:**
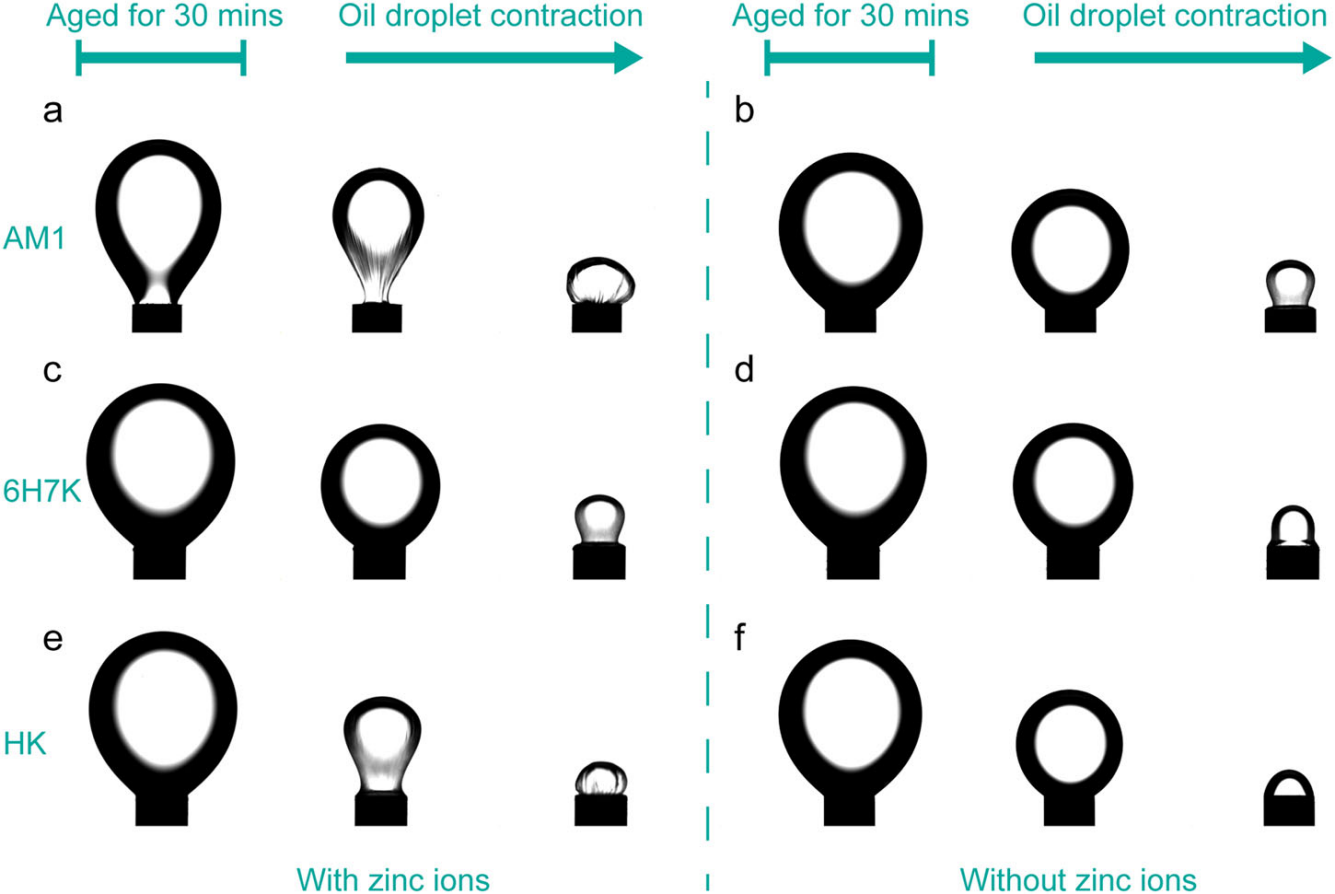
Visualization of the impacts of zinc ions at the oil/water interface. Shrinking effects of **a** and **b** AM1, **c** and **d** 6H7K, and **e**, **f** HK. The solution was prepared with a peptide concentration of 100 µM, with (200 µM zinc chloride) and without zinc ions. 80 µL for AM1 or 100 µL for heptads oil was dropped into an 8 mL peptide solution and the droplet was allowed to stabilize for 30 minutes. Images were taken in a row including after the droplet was stabilized for 30 minutes (first image), when it first started to shrink for the comparison of the volume (second image), and at the minimal volume of oil left (last image). The syringe diameter is 2 mm.

All the peptides in this study were designed to interact with metal ions through His-zinc coordination. To visualize the formation of an interfacial film as a result of His-zinc crosslinking at the oil-water interface, a shrinking experiment was performed. An oil droplet in peptide solutions was allowed to settle for 30 minutes, ensuring an equilibrium of adsorption and desorption rates. Subsequently, the oil droplet underwent a sudden volume reduction by suction to monitor its morphology change. In the presence of Zn^2+^, a wrinkled surface was observed for an oil droplet in the AM1 solution (Fig. 6a). As the contraction proceeded, a deflated balloon-like structure was formed with the decrease of the droplet volume. In contrast, the surface of the oil droplet remained smooth in the absence of Zn^2+^ in the AM1 solution (Fig. 6b). This indicates that a strong interfacial network was formed due to the His-zinc interaction in the presence of zinc ions^37^.

For 6H7K heptad, a slightly wrinkled surface was formed in the presence of Zn^2+^ ions at a smaller volume of oil droplet (Fig. 6c), suggesting the formation of a weak interfacial film and a smooth droplet was observed in the absence of Zn^2+^ (Fig. 6d). Compared to 6H7K, a slightly stronger interfacial network was generated at the surface of oil droplet surrounded by HK peptide solution as shown by the crumbled surface at a relatively bigger volume (Fig. 6e). The droplet remained smooth for HK in the absence of Zn^2+^ (Fig. 6f). These results demonstrated that both 6H7K and HK exhibited similar metal ion responsive behavior as that of AM1, and formed an interfacial network in the presence of metal ions.

The metal ion-responsive property of the emulsions was investigated using the HK peptide. Upon the addition of ethylenediaminetetraacetic acid (EDTA) to chelate the Zinc ions, the original emulsion with the particle size of 164.8 nm increased significantly to 596.4 nm with an obvious phage separation (Supplementary Fig. 3). Then by adding extra zinc ions to the unstabilized emulsion, good emulsions with a size of 170.6 nm can be re-generated under sonication, indicating the metal ion-responsive property is reversible.

### Characterization of peptide-stabilized nanoemulsions

Emulsions have a wide range of applications including pharmaceutics, foods, agriculture, etc^1–3^. Their interaction with other ingredients determines stability and function. For example, they are instantly diluted hundreds of times when injected into the bloodstream as medicines. Moreover, when characterizing emulsions using Dynamic Light Scattering (DLS), it is essential to measure with appropriate dilution. This ensures random Brownian motion and prevents multiple scattering. However, care must be taken as emulsions can become unstable upon dilution, potentially affecting the accuracy of the results.

**Fig. 7:**
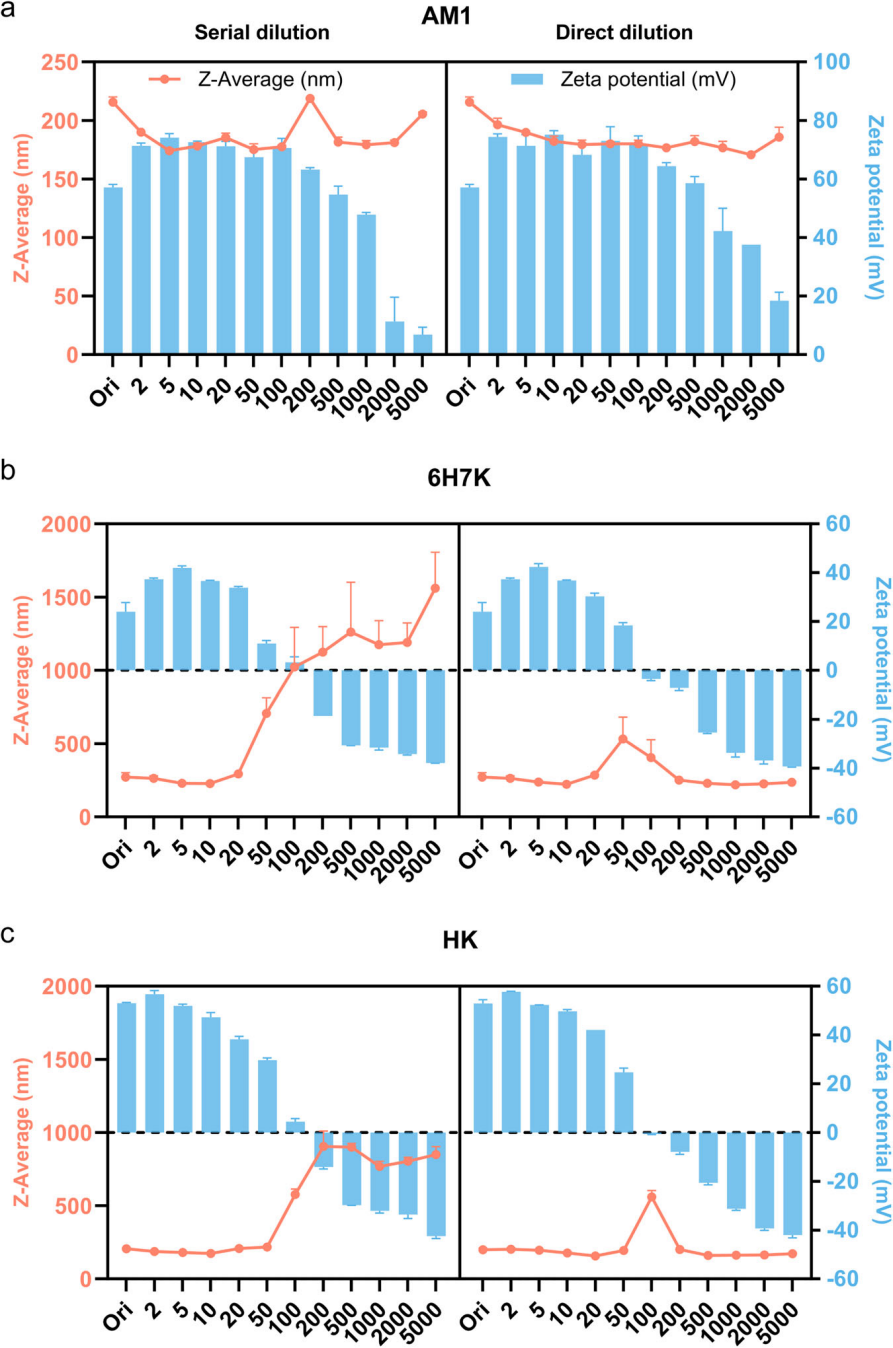
Optimization of the number of dilutions for peptide emulsions. **a** AM1, **b** 6H7K, and **c** HK. Peptide emulsions were made with a concentration of 267 µM for AM1, and 800 µM for 6H7K and HK, with zinc chloride at twice the concentration of the peptides, and 2% oil in 2.5 mM HEPES. Their droplet sizes (line graphs in red) and zeta potentials (bar charts in blue) were recorded. Error bars indicate the standard deviation of the z-averages and zeta potentials measured three times.

To optimize the number of dilutions, peptide emulsions were prepared in 2.5 mM HEPES and diluted by 2 to 5000 × into the solution with the same buffering background to compare droplet size and zeta potential. Two different types of dilutions were performed including serial dilution and direct dilution. The size of AM1 emulsions remained relatively stable at around 170–220 nm across all range of number of dilutions (Fig. 7a). Compared to the serial dilution, the droplet size was more stable when emulsions were diluted directly by the target number of dilutions. Overall, the zeta potential of the AM1 emulsion was positively charged and reversely related to the dilution numbers.

For 6H7K, its nanoemulsions exhibited a dilution-dependent size behavior in both serial dilution and direct dilution (Fig. 7b). For the serial dilution, the droplet sizes increased rapidly from 20 × dilutions, indicating the coalescence of droplets. This significant increase was mainly due to the reduced surface charge of the droplet, which fell below 20 mV and approached almost zero with 50 times and 100 times dilutions. Similarly, for direct dilution, a size increase was also observed at 50 and 100 times dilution due to the insufficient net charge. Upon further dilution, the emulsions become negatively charged. The difference between serial dilution and direct dilution lies in the rapid charge reverse of direct dilution upon high dilution times (e.g. > 200 times). Compared to serial dilution with irreversible coalescence, the reversed surface charge of direct dilution provided sufficient electrostatic repulsion between emulsion droplets, thereby ensuring their stability. The big error bars in Fig. 7b reflected the poor stability of the nanoemulsions after the 20 × dilutions for continuous dilution of 6H7K emulsion, so no consistent results could be obtained. The HK exhibited similar properties to those of the 6H7K, with a slight right shift in the critical dilution numbers (Fig. 7c). For HK, a significant increase in droplet size occurred at about 100 times dilution, where the net charge fell below 10 mV.

Comparing these three peptide surfactants, it is clear that AM1 is less likely to be affected by the dilution effects as they form a stronger film at the interface through His-zinc crosslinking, enabling them to anchor on the surface of the emulsion droplet tightly. In contrast, 6H7K and HK molecules are more dynamic, and their emulsion properties are prone to dilutions due to their smaller sizes. A sudden reduction in the peptide concentration in the surrounding solution of the emulsion droplets results in their desorption from the surface, leading to their instability as well as their reduced surface charge.

### Emulsion stability

**Fig. 8:**
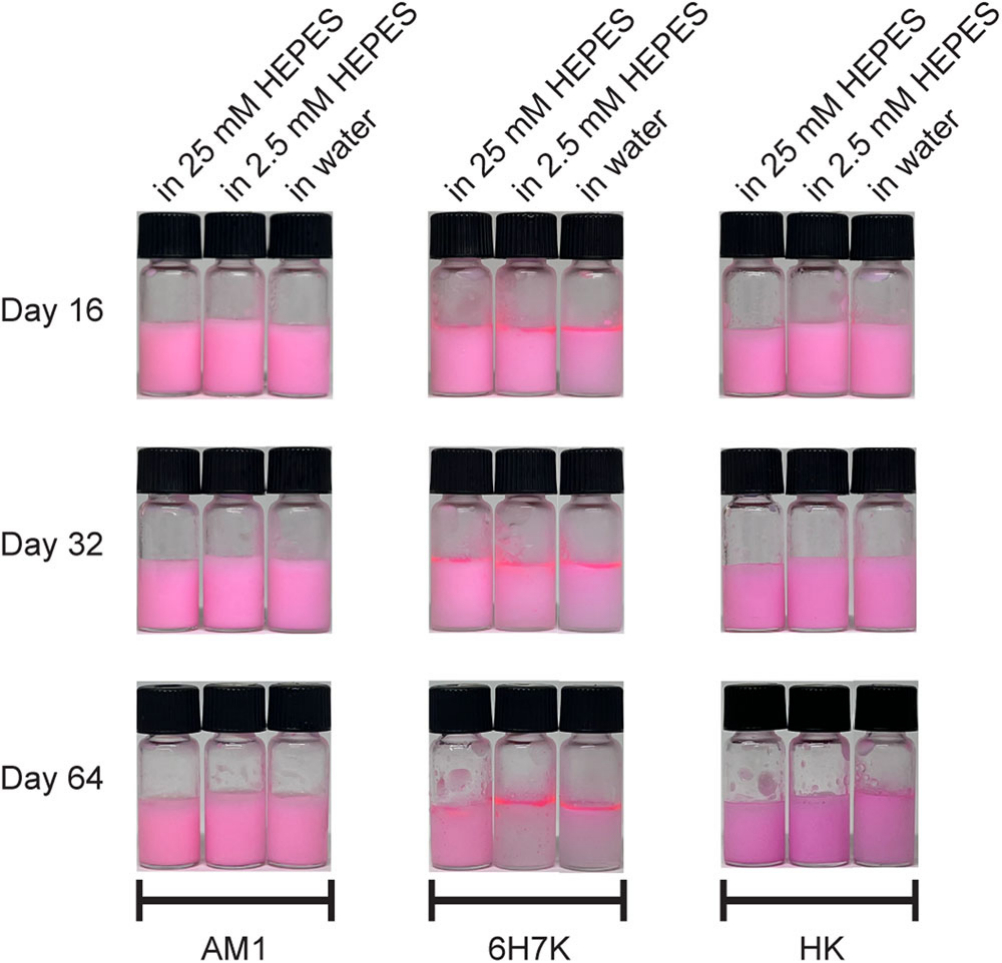
Stability of AM1,6H7K and HK emulsions over 64 days. Images of AM1, 6H7K, and HK emulsions were taken continuously after eight days on day 16, day 32, and day 64.

Following the initial experiments of the emulsion stability test (Fig. 1), the long-term stability of AM1, 6H7K, and HK emulsions in 25 mM HEPES, 2.5 mM HEPES, and water was continued to be monitored at 16, 32, and 64 days (Fig. 8). As previously discussed, 6H7K emulsions prepared in 25 mM HEPES, 2.5 mM HEPES, and water remained stable for the first four days, but started to show a slight phase separation at day 8 and completely separated at day 64. In contrast, AM1 and HK emulsions maintained stability throughout the entire 64-day monitoring period, showing no signs of phase separation.

**Fig. 9:**
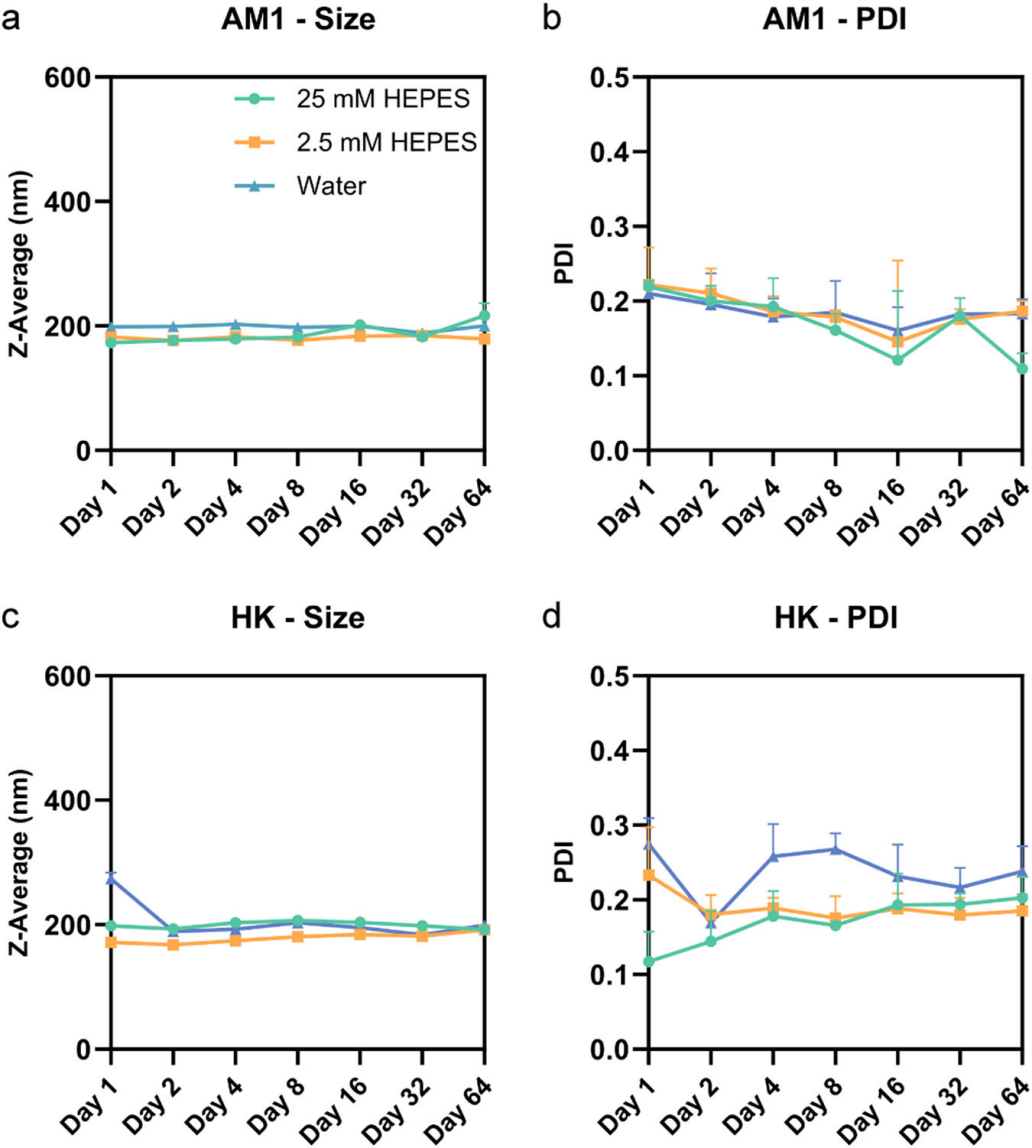
Size and size distribution of AM1 and HK emulsions over 64 days. **a**, **c** Sizes and **b**, **d** PDIs were measured on day 1, day 2, day 4, day 8, day 16, day 32, and day 64. Emulsions were made with a concentration of 800 µM HK and 267 µM for AM1 in 25 mM (spheres in green) and 2.5 mM HEPES (blocks in yellow) and water (triangles in blue). Zinc chloride at twice the concentration of the peptides was added to make all the emulsions. Error bars indicate the standard deviation of the z-averages and PDIs measured three times.

**Fig. 10:**
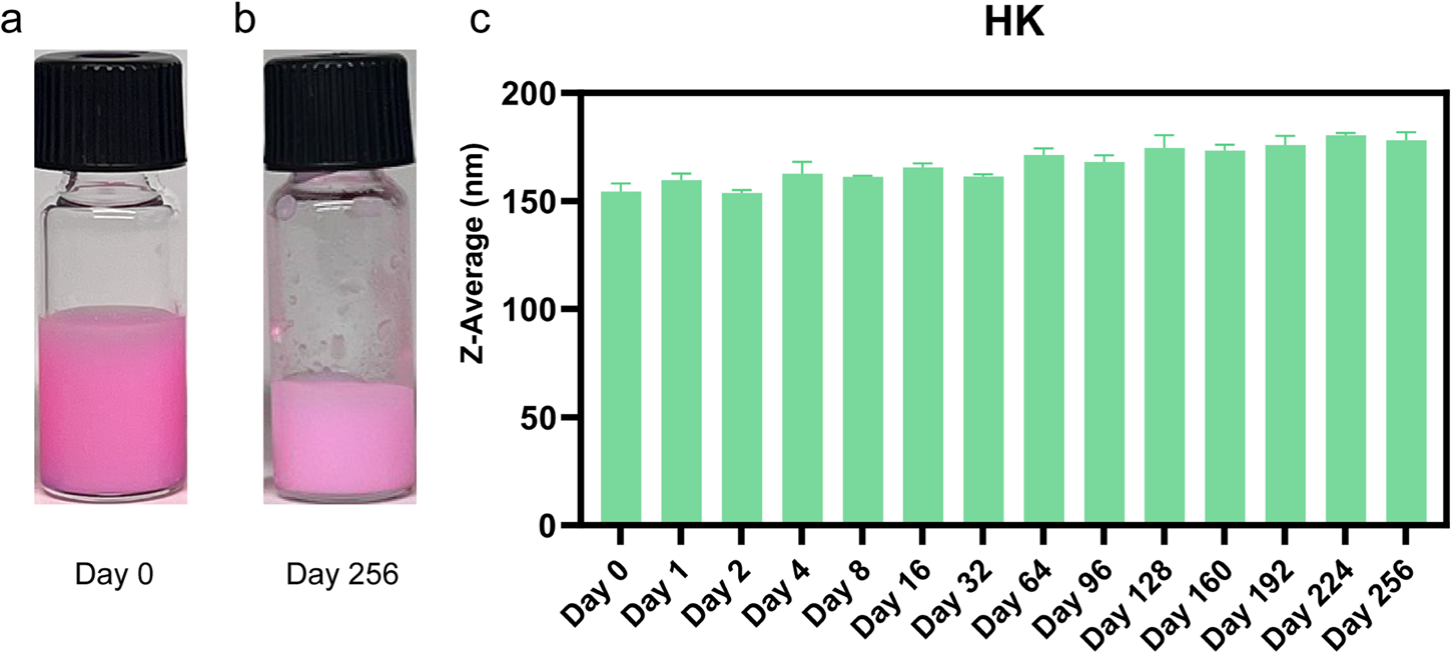
Stability of HK emulsion was monitored for 8 months. HK emulsion was made in 2.5 mM, pH 7.5 HEPES buffer with a peptide concentration of 800 µM and 1600 µM zinc chloride. Photos of the emulsion were taken **a** right after being made (day 0), and **b** 8 months after (day 256). **c** The size of the emulsion was continuously measured in water. Error bars indicate the standard deviation of the z-averages measured three times.

AM1 emulsions prepared in 25 mM HEPES, 2.5 mM HEPES, and water exhibited good stability, maintaining sizes between 173.1 and 202.9 nm (Fig. 9a) with PDIs below 0.3 (Fig. 9b). This stability can be attributed to the combined effects of electrostatic repulsion mediated by zeta potential and strong His-zinc coordination at the oil-water interface. HK emulsions also displayed consistent stability throughout the study, with sizes ranging from 167.6 to 273.8 nm (Fig. 9c) and PDIs consistently below 0.3 (Fig. 9d). The sizes and PDIs of 6H7K were measured and summarized in Supplementary Fig 4. The stability of AM1 (Supplementary Fig. 5) and HK (Fig. 10) emulsion made in 2.5 mM HEPES buffer at pH 7.5 remained stable for over 8 months.

Although HK and 6H7K have higher interfacial tensions compared to AM1, they are capable of stabilizing nanoemulsions for over 4 days and 8 months, respectively, with a stable size. This is mainly due to the positive net charge of the nanoemulsions. Zeta potential of the original emulsion samples stabilized by AM1 peptides had a positive value of 57.1 mV, while that of HK and 6H7K emulsions was measured to be + 52.9 and + 24.0 mV, respectively. These high net surface charges, in particular for HK emulsions, led to strong electrostatic repulsion, thus stable emulsions^8^. Due to the lower charge, the long-term stability of 6H7K emulsions was not as good as AM1 and HK (Fig. 8), showing a clear phase separation on Day 8.

In addition to electrostatic repulsion, another stabilization mechanism is steric hindrance which acts as a physical barrier preventing droplet interaction^38^. Due to the smaller size of heptads, they are less likely to exert steric effect. Thus, they are more susceptible to environmental factors such as buffers, and dilution factors (Fig. 9).

## Conclusions

In conclusion, this research paper provides insights into the design of peptide surfactants adopting a single heptad following a side-by-side structure at the interface, assessing and optimizing their performance in making nanoemulsions. Inspired by the previous work of our group, we took one heptad sequence from the AM1 peptide and modified it to create four heptad surfactants, AM1.2, 6H, 6H7K, and HK. Notably, HK was capable of stabilizing the emulsions in both HEPES buffer and water for over 64 days attributed to His-zinc coordination verified by the contraction experiment. We further explored the impacts of buffering salts, ionic strength, and the number of dilutions to guide future peptide surfactant design. In this study, buffer strength plays an important role in emulsion stability, a lower ionic strength buffer like HEPES is suitable for making stable emulsions. Additionally, it is important to pay attention to the characterization of emulsions using DLS, proper dilution numbers should be determined before conducting DLS measurement. As the dilution affects not only the stability of the emulsion, but also the adsorption-desorption kinetics of surfactant molecules on the interface. Nevertheless, very stable emulsions can be achieved by designing a single-heptad peptide surfactant in combination with the metal-ion mediated interfacial cross-linking. The HK peptide demonstrated long-term emulsion stability for up to 8 months.

## Methods

### Materials

Peptides were synthesized by GenScript with a purity greater than 95% confirmed by high-performance liquid chromatography. All peptides were dissolved in Milli-Q, aliquoted, and then lipolyzed using Lyophilizer (Christ-Alpha 1-2 LSCbasic). HEPES and PBS were purchased from Sigma Aldrich with a purity greater than 99.5% to prepare buffers. Mig 812 N was purchased from IOI Oleochemical. Hydrochloric acid was purchased from Merck & Co with a purity of 37% to prepare a hydrochloric acid solution with a concentration of 1 M to adjust the pH. Sodium hydroxide was purchased from ChemSupply to prepare a 1 M solution to adjust the pH. Zinc chloride was purchased from Sigma Aldrich with a purity of greater than 98% to prepare the stock solution with a concentration of 80 mM in Milli-Q. Milli-Q water with a resistivity greater than 18.2 MΩ•cm filtered by 0.22 µm filter (Merck, Bayswater, Australia) was adopted for all experiments. DiI Stain (1,1’-Dioctadecyl-3,3,3’,3’-Tetramethylindocarbocyanine Perchlorate) was purchased from Sigma-Aldrich with a purity of ≥ 98.0% TLC to help visualize the stability of peptide emulsions. Silicone oil with a viscosity of 20 cSt, and glycerol with a purity > 99.5% were purchased from Sigma Aldrich for Circular Dichroism measurement. Zinc sulfate (heptahydrate) was purchased from Chem-Supply. EDTA was purchased from Sigma Aldrich.

### Dynamic surface tension

A drop shape analyzer − 25S SN30014173 (Kruss) was employed to measure the dynamic surface tension under room temperature. Lyophilized peptide samples were dissolved in target buffer with a final concentration of 100 µM. To allow the His-zinc coordination, an excessive zinc chloride stock solution was added to the peptide solution with a final concentration of 200 µM. 80 µL for AM1 or 100 µL for heptads Mig 812 N was injected through a U-shaped needle into a cubic quartz cuvette containing 8 mL of the target peptide solution. Surface tension was monitored throughout the experiment with a duration of 600 s and the data was recorded every second.

### Shrinking experiment

The shrinking experiment was conducted using the same setup as measuring interfacial tension. 100 μM peptide solutions were prepared with (200 μM) and without zinc chloride. An oil droplet of a volume of 80 µL for AM1 or 100 µL for heptads was formed in a peptide solution of 8 mL in a cuvette and then allowed to stabilize for a minimum of 30 minutes to ensure equilibrium at the oil-water interface. After that, the oil droplet was sucked back into the syringe suddenly and shrunk to a much smaller volume. The morphology change of the droplet was recorded during the droplet contraction.

### Emulsion preparation

Lyophilized heptads (AM1.2, 6H, 6H7K, and HK) were first dissolved in a buffer solution at a concentration of 800 µM. A small amount of zinc chloride stock solution was added to the peptide solution to a final concentration of 1600 µM. The AM1 peptide solution was prepared with one-third of the molar concentration (267 µM) of heptads, as it consists of three heptad repeats. All emulsions were prepared with a 2% oil volume of Mig 812 N in HEPES or PBS buffers with a targeted concentration of pH 7.5. DiI was added to Mig 812 N with a stock concentration of 0.5 mg mL^−1^ to help visualize the emulsions. Emulsions stabilized by HK were made with 2% Mig 812 N (labeled with and without DiI) to investigate the effects of DiI to emulsion properties (Supplementary Fig. 1). The peptide solution was then mixed with oil with a volume of 1 mL and mixed with a probe sonifier (Branson digital sonifier – SFX550) for 30 s and 5 runs at a vibration output of 10 %. Emulsion samples were then diluted by the different number of dilutions in a target buffer for characterization and stability studies.

### Characterization of nanoemulsions

The hydrodynamic particle sizes and ζ potentials of the prepared emulsions were measured by DLS using a Malvern Zetasizer Nano ZS (Malvern Instruments, Malvern, UK) at 22 °C. The stability of the emulsions was monitored over 64 days with sizes and ζ potentials recorded on the set time points.

### Metal ion responsive behavior

The metal ion-responsive behavior of a nanoemulsion stabilized by the HK heptad peptide was studied. A stable emulsion was prepared using a peptide concentration of 800 µM and a zinc chloride concentration of 1.6 mM, with particle size measured by dynamic light scattering. Excessive EDTA was then added to chelate the zinc ions with a final concentration of 1.8 mM, followed by particle size measurement. Subsequently, zinc chloride was added to the emulsion with a final concentration of 2 mM, followed by a second sonication to assess the reversibility of the process.

### Structure investigation

Jasco (J − 815) CD was used to validate the secondary structure of peptides in bulk solutions, based on the methods published in previous studies^33,34,39^. Basically, the peptide solution was filled in a 500 µL quartz cell at 20–25 °C. UV CD spectra in the wavelength range of 190–260 nm were recorded using 0.1 nm data pitch, 50 nm s^−1^ scan speed, 2 s response time, 1 nm bandwidth, and 10 accumulations. Two data sets were obtained including ellipticity against wavelength and photo-multiplier voltage (HT) versus wavelength.

To investigate the structure of peptides in bulk solution, peptide solutions of AM1, 6H7K, and HK with a concentration of 800 µM were prepared without zinc. Then it was diluted using 2.5 mM HEPES buffer into different concentrations in a range of 10 to 100 µM to optimize the CD performance. The peptide concentration was optimized with HT values adjusted to sitting close to or below 600 according to Harvard J-815 Spectropolarimeter Starter Guide^40^ to avoid noisy results. Based on the preliminary results, 80 µM was selected for heptads, and 40 µM was selected for longer AM1 peptide as it becomes noisy when a higher concentration was used. Samples were prepared with and without zinc sulfate to compare their secondary structures. Two times the peptide concentration was used to allow excessive zinc ions to His amino residues.

The interfacial structures of peptides were studied based on methods reported in previous studies^32,35,36^. Silicone oil and glycerol were purchased from Sigma Aldrich. Silicone oil was used to make emulsions as it has a refractive index of 1.403, which is lower than Mig 812 N (RI = 1.450). To adjust the refractive index of the water phase to be the same as the oil phase, glycerol (RI = 1.47) was used. Emulsions were made in 2.5 mM HEPES, pH 7.5 with a peptide concentration of 800 µM and 1600 µM of zinc chloride. All emulsions were dialyzed in Milli-Q water with a volume of > 500 times the emulsion volume to minimize the chloride concentration. We tested the emulsions with 2% and 10% Mig 812 N and proceeded with 2% oil to minimize the noise. The concentration of emulsions was optimized. Firstly, we used the same peptide concentration (66 µM) as the CD measurements in the bulk solution. It works perfectly for AM1. We tripled the concentration used to measure heptad structure at the interface.

### Molecular dynamics simulation

Classical MD simulations were executed using the open-source GROMACS package^41^ in conjunction with the CHARMM36 force field^42^. The topological files and force field parameters were derived from the CGENFF web service (https://cgenff.com/)^43^, with the Cyclohexane (e.g., oil) molecule exhibiting both parameter and charge penalty of 0, suggesting a perfect parameterization and charge analogy with existing configurations parameterized by the CHARMM36 force field. This indicates that no further validation or optimization of the force field parameters is necessary.

The initial simulation setup featured a peptide LHQLAHK molecule solvated at the interface of 8184 water molecules and 1258 oil molecules. The simulation box dimensions were set as 90 Å × 90 Å × 60 Å, yielding a water density of approximately 1 g cm^−3^ and oil density of 0.72 g cm^−3^. To counterbalance the charge of the one positive charge of the peptide, one chlorine ion was introduced. The simulation protocol commenced with energy minimization over 50,000 steps using the steepest descent algorithm^44^, followed by a 20 ns constant number of particles, volume, and temperature (NVT) ensembled simulation (T = 300 K), and a subsequent 1000 ns constant number of particles, pressure, and temperature (NPT) ensembled simulation (P = 1 atm, T = 300 K), both with a time step of 2 fs. During the NVT and NPT phases, bonds involving hydrogen atoms were constrained using the LINCS algorithm^45^ with an order of 4. Temperature regulation at 300 K was achieved using the v-rescale thermostat, while pressure control was managed by the Berendsen developed approach^46^ with coupling constants set to 2.5 ps for both temperature and pressure. The Fast Smooth Particle-Mesh Ewald method^47^ was employed for the accurate and efficient treatment of long-range electrostatics and van der Waals interactions. The resulting configurations after the 1000-ns NPT simulations were processed using PyMOL^48^.

## Supplementary information

Supplementary Information

## Data Availability

The authors declare that the data supporting the findings of this study are available within the paper and its supplementary information files. Further data supporting the findings of this study are available from the authors upon reasonable request.
