# Supplementary material for: Design of stimuli-responsive minimalist heptad surfactants for stable emulsions
Source: Commun Mater. 2024 Oct 15;5(1):229. doi: 10.1038/s43246-024-00670-6 (PMC11473402; doi:10.1038/s43246-024-00670-6)
Supplement: Supplementary file 1 — Supplementary Information [file 43246_2024_670_MOESM1_ESM.pdf]

Supplementary information

Design of stimuli-responsive minimalist heptad surfactants for stable emulsions

Yang Li [1,2,3], Yilun Weng [3], Yue Hui [1], Jiaqi Wang [4], Letao Xu [1,3], Yang Yang [1,2], Guangze Yang [1,2*], Chun-Xia Zhao [1,2*]

[1] School of Chemical Engineering, Faculty of Science, Engineering and Technology, The University of Adelaide, Adelaide, SA 5005, Australia.
[2] ARC Centre of Excellence for Enabling Eco-Efficient Beneficiation of Minerals, Australia.
[3] Australian Institute for Bioengineering and Nanotechnology, The University of Queensland, Brisbane, QLD 4072, Australia.
[4] Wisdom Lake Academy of Pharmacy, Xi'an Jiaotong - Liverpool University, Suzhou, Jiangsu 215123, China.

* Corresponding authors
guangze.yang@adelaide.edu.au
chunxia.zhao@adelaide.edu.au

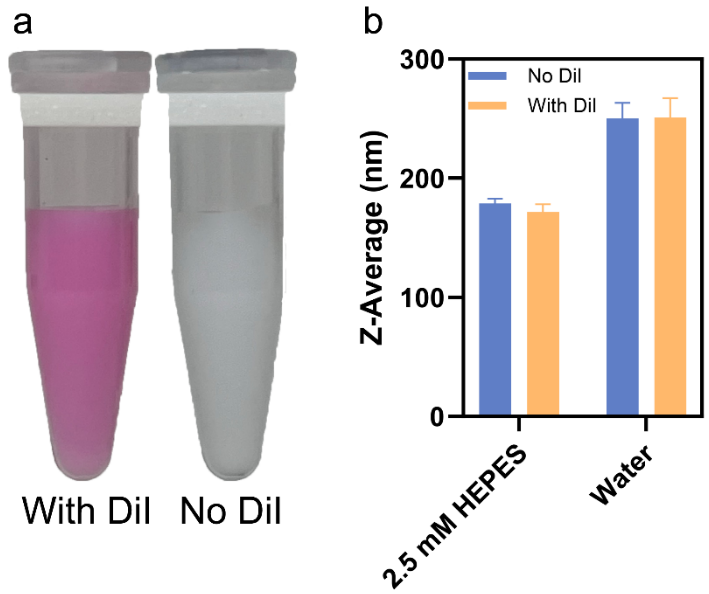

Supplementary Figure 1. Investigation on impacts of DiI in making emulsions. **a** HK heptad were employed to stabilize the emulsions using 2% Mig 812N (labeled with and without DiI) in the same buffer condition. **b** The emulsions were then diluted 500 times in 2.5 mM HEPES, and water to study their sizes. Error bars indicate the standard deviation of the Z-Averages measured three times.

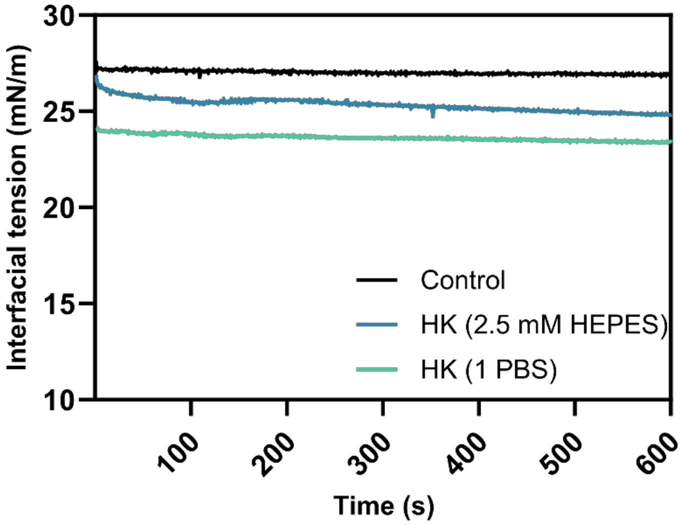

Supplementary Figure 2. Dynamic interfacial tension measurements of 100 µM HK heptad in the presence of 200 µM zinc chloride when dissolved in different buffers, 2.5 mM HEPES (blue), and 1 × PBS (green).

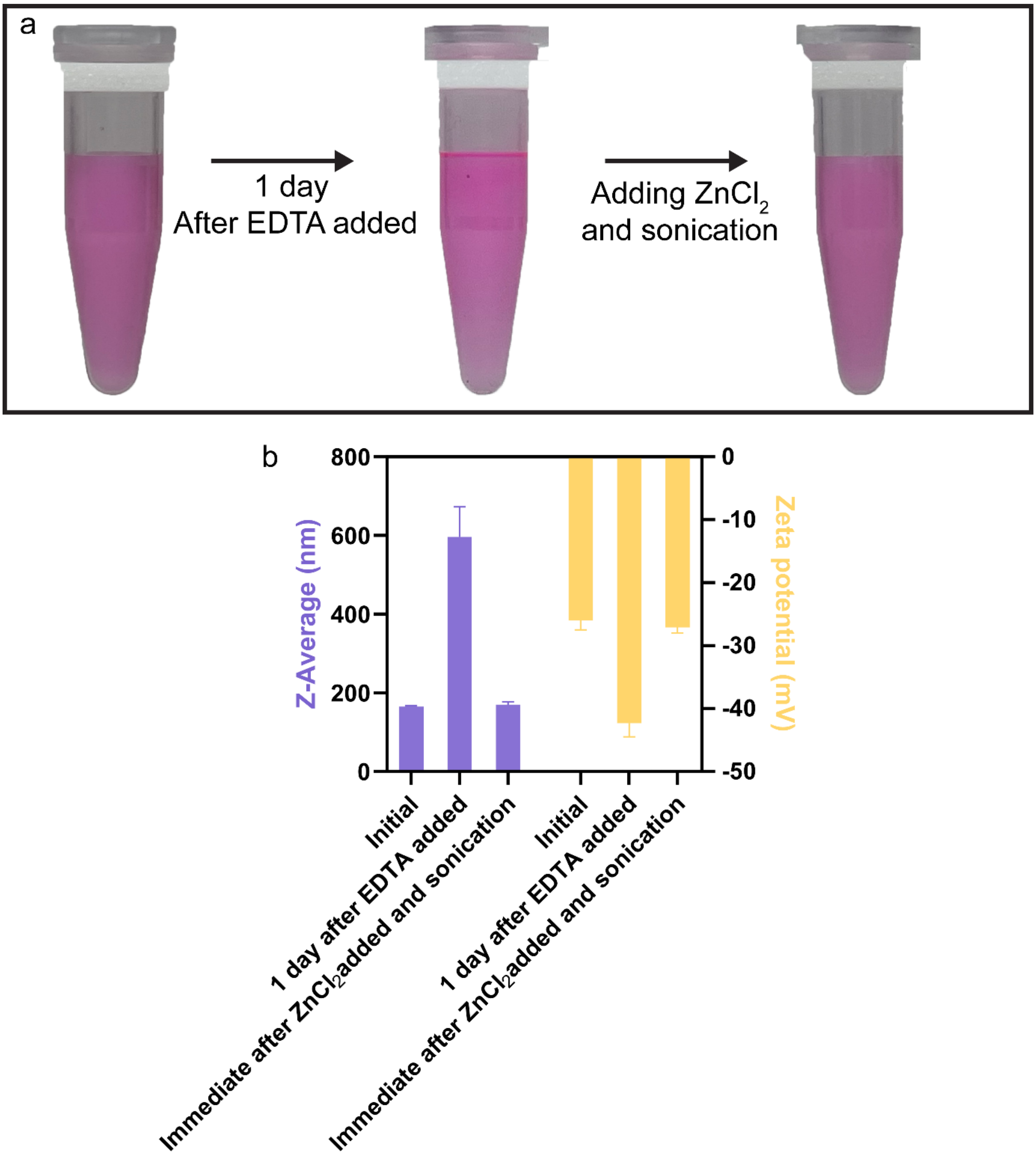

Supplementary Figure 3. **a** Images of HK emulsion were made with a peptide concentration of 800 µM and 1600 µM zinc chloride, 1 day after EDTA solution was added to the emulsion with a final concentration of 1800 µM and zinc chloride was added to the emulsion with a final concentration of 2000 µM. **b** their sizes and Zeta potentials were measured and compared. Error bars indicate the standard deviation of the Z-Averages and Zeta potentials measured three times.

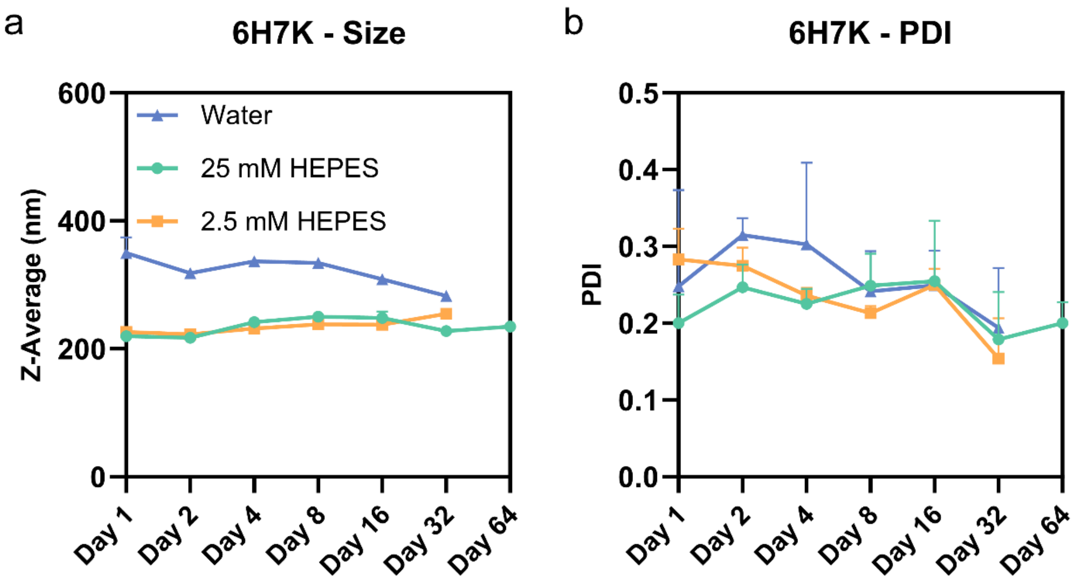

Supplementary Figure 4. Size and size distribution of 6H7K emulsions over 64 days. **a** Sizes and **b** PDIs were measured on day 1, day 2, day 4, day 8, day 16, day 32, and day 64. Emulsions were made with a concentration of 800 µM for 6H7K in 25 mM (spheres in green) and 2.5 mM (squared in yellow) HEPES and water (triangles in blue). Zinc chloride at twice the concentration of the peptides was added to make all the emulsions. Error bars indicate the standard deviation of the Z-Averages and PDIs measured three times.

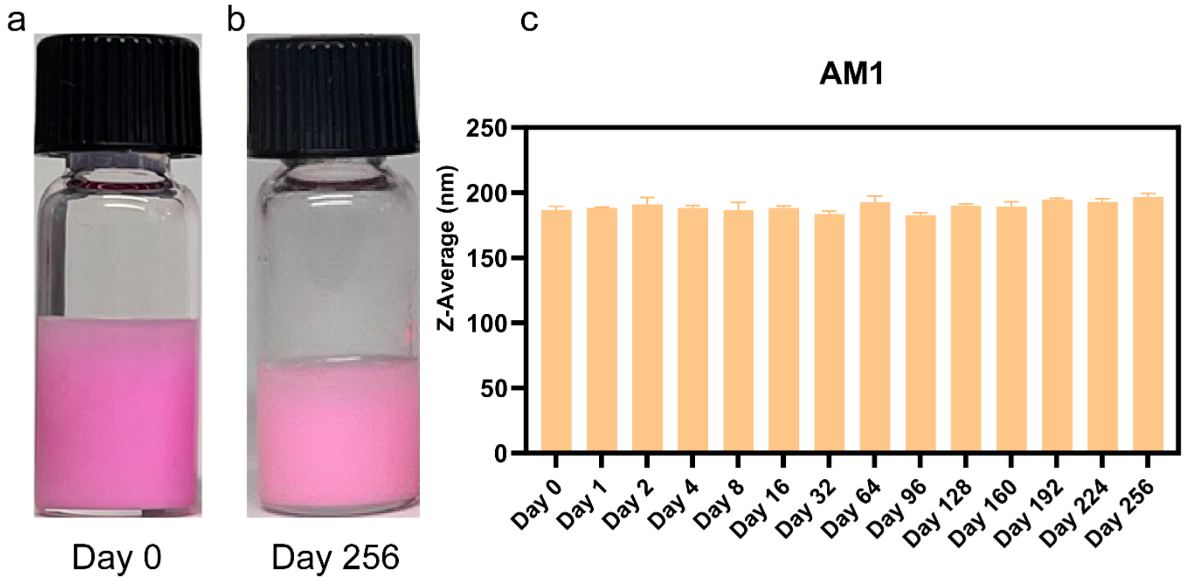

Supplementary Figure 5. Stability of AM1 emulsion was monitored for 8 months. AM1 emulsion was made in 2.5 mM, pH 7.5 HEPES buffer with a peptide concentration of 800 μM and 1600 μM zinc chloride. Photos of the emulsion were taken **a** right after being made (day 0), and **b** 8 months after (day 256). **c** The size of the emulsion was continuously measured in water. Error bars indicate the standard deviation of the Z-Averages measured three times.
